# Biological Macromolecule-Based Dressings for Combat Wounds: From Collagen to Growth Factors—A Review

**DOI:** 10.3390/medsci13030106

**Published:** 2025-08-01

**Authors:** Wojciech Kamysz, Patrycja Kleczkowska

**Affiliations:** 1 Department of Inorganic Chemistry, Faculty of Pharmacy, Medical University of Gdansk, 80-416 Gdansk, Poland; kamysz@gumed.edu.pl; 2 Department of Biomedical Research, National Medicines Institute, 00-725 Warsaw, Poland; 3 Maria Sklodowska-Curie Medical Academy in Warsaw, 03-411 Warsaw, Poland

**Keywords:** wound dressing, healing properties, efficacy, combat injuries, benefits

## Abstract

Wound care in military and combat environments poses distinct challenges that set it apart from conventional medical practice in civilian settings. The nature of injuries sustained on the battlefield—often complex, contaminated, and involving extensive tissue damage—combined with limited access to immediate medical intervention, significantly increases the risk of infection, delayed healing, and adverse outcomes. Traditional wound dressings frequently prove inadequate under such extreme conditions, as they have not been designed to address the specific physiological and logistical constraints present during armed conflicts. This review provides a comprehensive overview of recent progress in the development of advanced wound dressings tailored for use in military scenarios. Special attention has been given to multifunctional dressings that go beyond basic wound coverage by incorporating biologically active macromolecules such as collagen, chitosan, thrombin, alginate, therapeutic peptides, and growth factors. These compounds contribute to properties including moisture balance control, exudate absorption, microbial entrapment, and protection against secondary infection. This review highlights the critical role of advanced wound dressings in improving medical outcomes for injured military personnel. The potential of these technologies to reduce complications, enhance healing rates, and ultimately save lives underscores their growing importance in modern battlefield medicine.

## 1. Introduction

The wound can be defined as an interruption of anatomical continuity of the skin and mucous membranes accompanied by cellular damage and injured or pathological tissues [1]. While this definition applies broadly, the complexity of wound care increases substantially when contextualized within varying clinical and environmental conditions. Wounds may arise from diverse etiologies—including mechanical trauma, burns, chemical exposure, or surgical intervention—and are commonly categorized based on factors such as the cause, the extent of tissue involvement, the healing process, and the degree of contamination. Accordingly, wounds can be categorized as simple or complex; thermal, chemical, mechanical; and as either acute or chronic ones [2]. This classification becomes significantly more complex when considering the wounds sustained on the battlefield and the specific conditions under which they occur. Combat-related wounds represent a distinct clinical category characterized by their high-energy origin, polytrauma potential, and frequent contamination. Wartime injuries differ markedly from those seen in civilian contexts, both in terms of their severity and their propensity for rapid deterioration due to delayed intervention and environmental exposure [3]. These injuries are often inflicted under hostile conditions, such as blast exposures, gunfire, shrapnel, or thermal events, and are commonly accompanied by significant soft tissue loss, devitalized tissue, and a heightened risk of infection from environmental or biological contaminants.

It has been well established that all wounds require prompt medical attention to prevent progression, further tissue damage, or even mortality. Obviously, under peacetime conditions, such treatment is typically administered without delay by trained medical personnel, ensuring effective care. In contrast, during armed conflict or combat situations, both time and resources are severely limited. Moreover, the wound microenvironment is often markedly altered [4], while many existing wound care products fail to adequately address the unique challenging posed in such scenarios. Conventional wound care materials often fall short in addressing the unique challenges posed by battlefield injuries. Most traditional dressings function passively, offering only basic protection without actively engaging in the healing process. Yet in combat settings, there is a critical need for dressings that are not only easy to apply under duress but are also multifunctional—capable of regulating moisture, absorbing exudate, preventing microbial infiltration, controlling hemorrhage, and potentially providing analgesia. Ideally, such a dressing should fulfill critical functions, including moisture regulation, absorption of wound exudate, and entrapment of microorganisms to prevent secondary infection. By providing this protective barrier, the dressing could significantly enhance healing activities.

In recent years, significant progress has been made in the design of advanced wound dressings that integrate pharmacologically active agents to accelerate healing and reduce complications. These next-generation materials include antimicrobial and analgesic agents that aim to control infection, manage pain, and support the body’s natural repair mechanisms. Moreover, innovations in biomaterials science have enabled the creation of smart dressings with responsive properties, such as controlled drug release or environmental sensing capabilities.

This review aims to present recent advancements in the development of wound dressings that have emerged as vital tools in battlefield medicine. Special emphasis is placed on dressings incorporating pharmacologically active compounds—particularly those with antimicrobial and analgesic properties—and on the translational potential of these innovations for use in resource-constrained, high-risk environments such as the battlefield. By highlighting both technological achievements and unmet needs, this review seeks to inform future directions in combat wound care and the design of next-generation therapeutic materials.

## 2. Battlefield Wounds—Classification and Associated Challenges

Combat-related injuries encompass a wide range of wound types; however, their effective management remains a significant clinical challenge, despite ongoing advancements and considerable research efforts. Several classification systems have been proposed to better characterize and address the complexities of battlefield wounds. One notable approach is the classification outlined by Granick and Chehade [5], which categorizes wounds based on the extent and adequacy of debridement: (i) incomplete—where necrotic tissue remains, (ii) marginal—with partial removal of devitalized tissue, and (iii) complete debridement—where all non-viable tissues are removed. This classification provides clinicians with a practical framework to determine wound severity and select appropriate treatment strategies.

Another common method of classification pertains to the mechanism of injury, particularly those caused by explosive devices and firearms. Ballistic wounds, for instance, are further categorized as penetrating, perforating, or avulsive, depending on the trajectory, tissue damage, and presence of an exit wound [6]. Furthermore, blast injuries, prevalent in modern warfare, are systematically divided into four categories: (i) primary, (ii) secondary, (iii) tertiary, and (iv) quaternary ones. Each of these subtypes arises from distinct pathophysiological mechanisms [7,8,9]. For example, primary blast injuries are primarily caused by the direct effect of the blast wave on bodily tissues, whereas quaternary injuries result from associated thermal, chemical, or radiological exposures (for details, see [10]).

A simplified yet practical classification scheme, designed to facilitate rapid triage and treatment in resource-constrained or chaotic combat environments, has been proposed by the International Committee of the Red Cross (ICRC) [11]. This system stratifies injuries into three grades, reflecting increasing levels of severity, as illustrated in Table 1. Although not explicitly included in the original classification, the presence of metallic foreign bodies, detected via radiographic imaging, is often considered. This extension of the Red Cross system is sometimes referred to by the acronym EXCFMV, which incorporates key wound characteristics: Entry (E), Exit (X), Cavity (C), Fracture (F), involvement of Vital structures (V), and presence of Metallic bodies (M).

As compared to civilian injuries, combat-related wounds are markedly more complex and challenging to manage. One of the key contributing factors is the battlefield environment, which significantly increases the likelihood of wound contamination, particularly with microorganisms. Although most wounds become colonized by multiple pathogens simultaneously, the primary agents responsible for infection are typically Gram-positive cocci, notably *Staphylococcus aureus* and *Streptococcus pyogenes*, both known for their enzyme and toxin production. In addition, the Gram-negative, exopolysaccharide-producing *Pseudomonas aeruginosa*—a highly adaptable and virulent pathogen—poses a significant threat due to its aggressive colonization and toxin secretion [12]. Inadequate hygiene conditions, often inherent in combat scenarios, further contribute to the risk of infection, frequently precluding timely surgical reconstruction and primary closure [13]. Notably, infection of open combat wounds has been reported as relatively common, even when standard protocols such as prompt surgical debridement and prophylactic antibiotic administration are followed [14]. Furthermore, the severity of combat wounds is often compounded by their size and depth, as penetrating trauma predominates in warfare. These injuries frequently involve multiple anatomical regions and are commonly associated with extensive hemorrhage [15].

As a consequence, poor wound healing and antimicrobial resistance represent two of the most significant complications encountered in the treatment of severe extremity injuries sustained on the battlefield [16].

Another critical challenge unique to combat wounds is the inability to achieve timely wound closure. This is often due to a shortage of skilled personnel and the absence of specialized medical infrastructure in combat zones. Moreover, selection of appropriate wound dressings becomes crucial, particularly when immediate care must be provided under suboptimal conditions. Inappropriate use of dressing materials can exacerbate the infection, especially when foreign substances are introduced into already contaminated wounds. For example, hydrocolloid dressings, while beneficial in specific clinical situations, are generally contraindicated for use in burns (except for small partial-thickness burns), dry wounds, wounds with heavy exudate, tunneling wounds, sinus tracts, infected wounds, or wounds with exposed tendons, bones, or fragile periwound skin [17,18]. Another example includes oxidized regenerated cellulose, which, although being effective as a hemostatic agent, may cause compressive complications when applied near neural structures or within confined anatomical space [19]. Therefore, careful evaluation of the physical and biological properties of dressing materials is essential to ensure they support rather than hinder the wound healing process.

## 3. Wound Healing Dressings Incorporating Bioactive Compounds—Characterization and Benefits

Given that virtually all wounds—particularly those sustained in combat—are accompanied by significant pain, the incorporation of analgesic compounds directly into wound dressings represents a rational and potentially valuable approach. This strategy is especially relevant in battlefield conditions, where injured individuals may experience impaired consciousness, rendering oral administration of analgesics unfeasible. Moreover, intravenous administration may not always be possible due to logistical constraints or the severity of the trauma.

Importantly, many bioactive analgesics also exhibit antimicrobial activity, which is a critical advantage in treating battlefield wounds. These injuries are frequently subject to extensive contamination due to environmental exposure, thereby increasing the risk of infection. As such, the dual action of these compounds can play a significant role in mitigating both pain and microbial proliferation.

Another essential consideration in the design of advanced wound dressings for military use is the ability to control bleeding without the need for external compression. Hemostatic dressings have emerged as an important solution in this regard, particularly in scenarios where immediate professional medical assistance is inaccessible. These dressings are designed to be applied easily by either the injured individual or an untrained responder, ensuring rapid hemostasis under emergency conditions.

In view of these requirements, ongoing research efforts are focused on the development of multifunctional wound dressings that combine hemostatic, analgesic, and antimicrobial properties. Such dressings aim to accelerate wound stabilization, reduce the risk of infection, and provide immediate pain relief, thereby improving survival outcomes and facilitating early stages of healing in austere environments.

### 3.1. Hemostatic Materials in Wound Dressings—Characteristics, Benefits, and Limitations

Numerous materials have been characterized to date for their capacity to control hemorrhage, a function that is particularly critical in the management of battlefield injuries. These materials include both biologically derived and synthetic compounds, such as collagen, gelatine, fibrin, thrombin, and fibrinogen, as well as zeolite, kaolin, chitosan, and cellulose [20,21,22,23].

Collagen, a naturally occurring structural protein abundant in the extracellular matrix of the skin, has widely been recognized for its high biocompatibility and low immunogenicity. It demonstrated efficacy in promoting skin health and regeneration. For instance, Tanaka et al. [24] reported that oral administration of collagen peptides attenuated UV-B-induced skin damage. In wound management, collagen has been shown to facilitate platelet activation and aggregation, ultimately leading to fibrin clot formation at the injury site [25,26]. Furthermore, collagen supports keratinocyte migration and re-epithelialization, contributing to accelerated wound closure [27]. These benefits are consistent across different sources of collagen; for example, marine-derived collagen significantly enhanced wound healing in rabbit models within 11 days as compared to that of the controls [28]. Notably, scar tissue has been found to exhibit increased collagen density and larger fiber dimensions relative to healthy skin [29], underscoring collagen’s pivotal role in tissue remodeling and its continued prominence in advanced wound dressing formulations.

Thrombin, also known as activated Factor II or Factor IIa, is a serine protease essential to the regulation of hemostasis [30]. It plays a central role in platelet aggregation and in the release of pro-inflammatory and growth factors that drive the recruitment of immune cells and support tissue repair and remodeling [31,32,33]. Active thrombin generated at vascular injury sites has been shown to stimulate proliferation and migration of endothelial cells, fibroblasts, and vascular smooth muscle cells. These processes are closely linked with thrombin-induced chemokine release, such as MCP-1, which further enhances leukocyte infiltration and cytokine-mediated repair [34]. However, its therapeutic application is constrained by its short biological half-life of approximately 15 s, due to rapid enzymatic degradation [35].

Kaolin, also termed China clay, on the other hand, is one of the most versatile industrial materials (e.g., in the paint and plastic industry, ceramic industry, etc.). In the aspect of wound healing, it was found in 1958 to be involved in blood clotting stimulation through activation of Factor XII induced by negative charges on the kaolin surface [36,37]. Moreover, this material acts by absorbing water from the blood, thereby concentrating the platelets and thrombin in the blood [38].

Finally, chitosan (also known as (1 → 4)-2-amino-2-deoxy-β-D-glucan; CS), a linear basic polysaccharide obtained by deacetylation of the natural polysaccharide chitin, is one of the popular hemostatic materials. Its properties (Table 2) used in wound healing therapy include: (i) excellent biocompatibility and biodegradability [39]; (ii) moderate water absorption capacity, though weaker than for kaolin [40]; (iii) ability to promote hemostasis through aggregation of erythrocytes and platelets [41]; (iv) stimulation of granulation tissue formation and skin proliferation; and (v) inherent antibacterial activity [41,42].

However, despite their benefits, each of the aforementioned materials is associated with specific limitations that can hinder their clinical utility. For example, collagen is susceptible to degradation in infected wounds due to enzymatic activity of microbial collagenases and gelatinases, potentially contributing to chronic wound formation [25]. Zeolite, although effective in water absorption, induces an exothermic reaction that can cause burns and inflammatory responses (Table 2) [43]. Also, in the case of kaolin, some limitations exist. Trabattoni and colleagues [44] reported that dressings incorporated with kaolin seem to be insufficient when applied once and not repetitively. Moreover, its effectiveness depends on the wound’s blood flow, making it more useful in the treatment of high-flow arterial and venous wounds [45]. Also, kaolin has been shown to induce hyperalgesia in preclinical pain models, which is particularly undesirable in already painful traumatic injuries [46,47].

Given the complexity and variability of battlefield injuries, the development of hemostatic dressings with optimized safety and efficacy profiles is of paramount importance. Ineffective or improperly selected materials can not only impair wound healing but can also lead to severe complications, including adverse surgical outcomes and uncontrollable physiological responses. Hence, prompt and evidence-based selection of dressing components is essential to mitigate progression to non-healing or chronic wounds and to support effective hemorrhage control across a range of anatomical locations.

#### 3.1.1. Hemostatic Dressing Efficacy in Animal Models of Wound Healing—An Overview

Animal models have widely been utilized to evaluate the efficacy and wound-healing potential of hemostatic agents. However, replicating battlefield injuries, such as deep, penetrating, and irregular traumatic wounds, is challenging due to their variability and complexity. Standardized replication of those injuries in animal models is particularly difficult. While hemorrhage from extremity wounds can often be managed with direct pressure or tourniquets, massive bleeding from complex injuries like those to the chest or pelvis remains a significant challenge. Notably, such wounds were identified as a leading cause of death among American military personnel in Somalia and Afghanistan [48]. Despite their limitations, animal models remain a cornerstone of preclinical testing.

**Figure 1:**
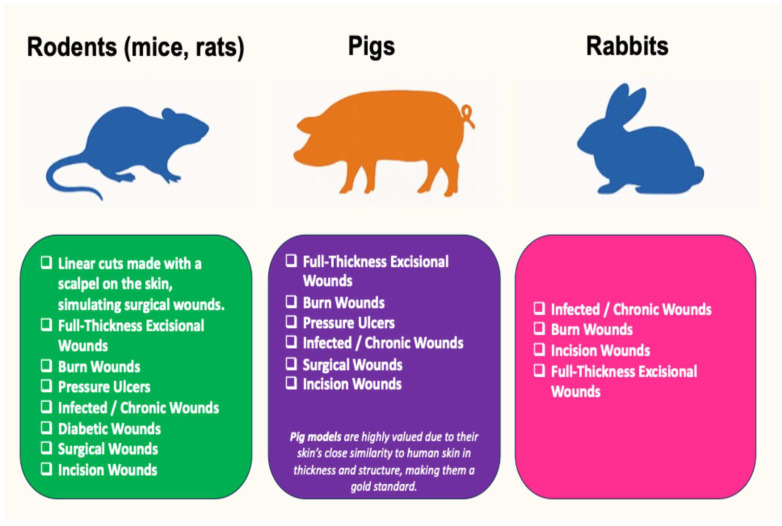
Animal models to assess hemostatic performance and wound healing properties of dressings.

Various animal models are employed in preclinical research to assess both the hemostatic performance and wound healing properties of novel dressings. Rodent tail or femoral artery transection models are commonly used to simulate peripheral bleeding and evaluate time to hemostasis. Liver or spleen injury models, particularly in rats or pigs, help investigate control of severe internal hemorrhage. For wound healing studies, full-thickness excisional or burn wound models in mice, rats, or rabbits are applied to evaluate epithelial regeneration, granulation tissue formation, and inflammation. Porcine models, due to their anatomical and physiological similarity to human skin, serve as a translational step before clinical trials (Figure 1).

Discrepancies in dressing effectiveness depending on wound type have been reported. For instance, Alam et al. [49] found that a hemostatic dressing containing microporous polysaccharide hemospheres was effective in accelerating clotting, in general, but failed to significantly reduce mortality in a femoral artery and vein transection model.

Similarly, while chitin-based dressings demonstrated fast coagulation in some studies [50], others found them no more effective than standard gauze in managing severe venous hemorrhage [51]. Also, Watters and colleagues performed a comparative study of several different types of dressings, including kaolin-based Combat Gauze (CG, Z-Medica, Wallingford, CT, USA) and a nonwoven chitosan-derived Celox Gauze (XG, SAM Medical Products, Wilsonville, OR, USA) [52]. The model used was a femoral artery injury one conducted in swine; however, with the introduction of the “care under fire” scenario. In this aspect, considering that all gauze-based hemostatic dressings require prolonged hold times (i.e., 2–5 min of compression), which is quite difficult to obtain under fire, the authors decided to perform the so-called “no-hold” model. The results indicated no significant differences between the dressings in terms of clot initiation time and total blood loss.

Aside from wound type and model, other biological variables, such as age, sex, and the animal’s microbiome, also influence outcomes [53,54]. Intriguingly, the hair growth cycle in animals can also differ from that of humans. In this aspect, it is widely implied that wounds in areas with higher hair density heal faster than those in less hairy or bald ones [55]. Hence, considering that some animals, such as rats/mice or rabbits, are characterized by hair on almost the entire surface of the body, the model used is likely to provide opportunities for different interpretations of the results in relation to those of the human body.

Despite problems that arise in recreating different types of battlefield wounds with animal models, making them the ultimate challenge, a plethora of such efforts is still being conducted to evaluate the efficacy and safety of potential therapeutic agents. For instance, in 1990, Raccuia et al. [56] determined the hemostatic efficiency of oxidized cellulose, collagen and fibrin glue in a rat kidney injury model and found that the fibrin glue has superior hemostatic ability compared to the other 2 materials [19]. Yoon et al. [19]. This group compared the absorbable fibrin patch Tachosil (Baxter, Deerfield, IL, USA), Surgicel Fibrillar absorbable oxidized regenerated cellulose (Johnson & Johnson, New Brunswick, NJ, USA) and absorbable oxidized regenerated cellulose Surgicel Snow (Johnson & Johnson) in an animal model of muscle damage to both rectus abdominis muscles in male Sprague Dawley rats. Wound healing was found to be faster in the fibrin-based material than in the other groups.

As has been said, fibrin appeared to be fairly effective in strongly and quickly conducting the healing process. Indeed, its efficacy is usually caused by its composition, i.e., fibrinogen, human thrombin, Ca^2+^, and factor XIII [19]. However, fibrin can act as a growth factor, directly affecting angiogenesis and promoting fibroblast formation [57], while the oxidized cellulose activity is thought to be indirect; it can modify chronic wound environments through a significant reduction in harmful factors such as proteases, whose excessive amount can lead to diminished growth factors’ activity and delayed healing [58,59].

In contrast, oxidized regenerative cellulose was found to be superior to the gelatin-based hemostatic agent (Spongostan) in an experimental penile fracture rat model [60]. It was claimed that cellulose dressing significantly enhanced primary wound healing and reduced fibrosis formation, which was absent in the case of Spongostan. Moreover, the latter was associated with an increased fibrosis during the healing process. Importantly, these results clearly demonstrate that the type of wound dressing can lead to various outcomes, which sometimes cannot be expected by the subject. In fact, as clearly stated by the authors, the development of fibrosis and scarring in penile tissue can result in complications such as curvature and erectile dysfunction [60,61]. Hence, with the above in mind, it is appropriate not only to choose the appropriate dressing depending on the type of wound but also to choose it based on the mechanism of action of the dressing and possible aesthetic effects.

One of the agents that recently has gained much interest is a montmorillonite clay, bentonite. In fact, owing to its properties, its different dressing compositions are expected to effectively remove toxins and protect against pathogenic infections caused by skin injury [62,63]. In this aspect, CoolClot is a type of hemostatic material developed in Iran, being composed of zeolite (1/3 by weight) and bentonite (2/3 by weight). Although the antibacterial effect was not measured, the zeolite-bentonite composition was found to moderately accelerate the wound healing process in a rat model of a full-thickness excisional circular skin wound. However, its topical application caused a significant increase in wound surface temperature (1.56 °C), thus indicating the role of zeolite [64], a mineral responsible for exothermic reaction and the consequent tissue burn.

Since plants have been in use since ancient civilizations to treat wounds of various origins [65,66,67,68], several authors have evaluated their potential as a component of hemostatic dressings [69,70]. In 2017 the group of Bouassida [71] revealed beneficial properties of *Urtica dioica* leaf extract dissolved in glycerol. The measurement was performed in a full-thickness excision wound model in Wistar rats. Moreover, the tip of rats’ tails was cut with a scalpel blade to cause bleeding and thus determine the bleeding time after application of the extract. As a result, it was found that the bleeding time was significantly reduced for the extract as compared to that of the untreated group or even the reference commercial cream. In addition, by day 11 of the treatment, almost all the wounds treated with *U. dioica* showed complete wound closure (92.39%) vs. only 85.36% of wound healing recorded by the control cream.

Another attractive example of the use of medicinal plants was announced by Sutar et al. [72]. Here, dressings based on pectin and alginate polymers coated with the crude extract of the *Croton oblongifolius* (CO) stem bark were presented. This composition offers desired properties, including a pectin-induced acidic environment at the wound site and the ability to bind growth factors, that result in efficient generation of new cells [73], improvement of absorption of the fluids from exuding wounds owing to the activity of alginate [74], and finally anti-inflammatory and antioxidant properties exerted by CO [75,76,77]. Unfortunately, the study has shown CO efficacy only in vitro, while to date there is no confirming information about its activity in vivo.

Nevertheless, there are a number of other scientific papers describing medicinal plants as useful components of various hemostatic materials. As reported by Ozturk and colleagues [78], application of mixed extracts of *Inula viscosa* and *Capsella bursa-pastoris*, termed IvCbp, was found to be attractive in a rat liver laceration model. In fact, animals treated with IvCbp had less blood loss, as confirmed by higher post-operative hemoglobin level, than that noticed in other groups.

Recently, evaluation of the healing potential of a novel wound dressing containing nanofibers with an ABS was reported by Sensoy et al. [79]. The ABS abbreviation is for the Ankaferd Blood Stopper, an antibacterial herbal extract composed of several active ingredients of dried roots and leaves of *Thymus vulgaris* L., *Glycyrrhiza glabra* L., *Vitis vinifera* L., *Alpinia officinarum Hance*, and *Urtica diocia* L. [79,80,81]. The authors demonstrated that ABS applied on a wound (15 mm in diam.) in female Wistar-albino rats resulted in healing acceleration, as reepithelization occurred at a high rate in contrast to that of control animals. Moreover, on the 14th day of the study, the wounds were completely closed in all rats treated with ABS [79]. These results are in agreement with others showing ABS involvement in epithelialization in a full-thickness skin wound model in rats [82,83].

Since alginate has been considered to be an excellent hemostatic polymer-based biomaterial owing to its excellent biocompatibility, biodegradability, non-toxicity, non-immunogenicity, easy gelation and easy availability [84], several authors have presented its efficacy in various models of wounds. One of the studies published in 2024 reported the use of alginate hydrogel dressings that contain silver nanoparticles (AgNPs), the antibacterial agent cefepime, and fibroblast growth factor-2 (FGF-2) in the treatment of an *S. aureus* + *P. aeruginosa*-induced purulent wound (of the gluteofemoral area and the withers) in male Wistar rats [85]. As a result, it was shown that such a complex, multifunctional dressing can easily and effectively induce comprehensive tissue regeneration by addressing both infection and regeneration.

Notably, the use of alginate hydrogel loaded with biologically active ingredients as a common hemostatic dressing presented here is not the first one. One of the examples was reported by Ehterami and colleagues [86], who determined the effect of a vitamin E-loaded hydrogel mixture of alginate and chitosan for the healing of the dorsal skin injury in a rat model (male Wistar rats). They indicated that Chit/Alg Hydrogel with Vit. E resulted in a wound with no signs of infection and inflammation in it; furthermore, the wound treatment was very effective. However, as noted in the paper, the beneficial effect strongly depended on the concentration of vitamin E. In fact, in a Chit/Alg hydrogel loaded with 1600 IU of vitamin E, the sign of infection appears after 14 days of treatment. Moreover, histopathologically, an epidermal proliferation was similar to that noticed in the group where no vitamin E was given. Importantly, the dried hydrogel or alginate can adhere to the wound bed and thus can injure the wound during its removal [87]. Unfortunately, the two articles mentioned above did not present any results in this context.

Importantly, among various elements used as active ingredients of the dressing, the activity of peptides and peptidomimetics was also evaluated. In this aspect, a 16-residue peptide RADARADARADARADA (RADA16-I). This self-assembled structure, composed of hydrophobic alanine and hydrophilic arginine (or aspartic acid), was found to reduce the blood loss in a kidney injury rat model (Sprague-Dawley male rats) when applied directly on the cut surface of the kidney at a concentration of 2%. In addition, this effect was similar to that of commercial Gelfoam, which was used as a control [88].

Right after the RADA16-I, some other peptides were discovered with improved properties. This refers to a self-assembled h9E peptide, which was developed by Sun and Huang in 2010 [89]. This novel structure combines two native protein domains: the β-spiral motif of the spider flagelliform silk protein and the calcium-binding motif of the human muscle protein. Importantly, when applied topically, it induced a concentration-dependent hemostatic effect in rats whose tail was cut off at a thickness of 5 mm. The best effect was obtained at a concentration of 5%.

Another attractive example of introducing peptides into battlefield medicine are elastin-based peptides with the sequence of [(CPGVG)_4_IPGVG]_14_ [90] or (SGVG([VPGVG]_2_VPGEG[VPGVG]_2_)_25_VPG) [91,92]. The interest in elastin has its own reasons. Indeed, it is an extracellular matrix component that controls the biocompatibility and biodegradability, as well as non-immunogenic characteristics, etc. [93]. However, when constructing protein-based dressings such as hydrogels, chemical crosslinking reactions stabilizing the hydrogels should be performed; otherwise, proteins or peptides will easily be degraded by various proteolytic enzymes. Moreover, in the aspect of chemical crosslinking, several toxic or active by-products can be obtained, which, in turn, definitely affect their safety profile and make it difficult to use them completely.

Other examples of wound hemostatic dressings and their effectiveness are shown in Table 3.

#### 3.1.2. Hemostatic Dressing Specifically Approved and Used in Battlefield Injuries and Austere Settings

As stated by Obagi et al. [100] an ideal wound dressing should possess three specific principles that include (a) providing a temporary protective physical barrier, (b) absorbing wound drainage, and (c) providing the moisture necessary to optimize re-epithelialization. Considering the battlefield environment, these criteria appear somehow to be difficult to meet. Indeed, in this case, the focus is on initial stabilization of the wound rather than definitive care and minimization of blood loss. Nevertheless, these principles also depend on the type of wound, as reported in the case of burn wounds by Shingleton et al. [101] and the group of Cancio [102].

Traditional, passive wound dressings include gauze and tulle, while the so-called modern wound care mainly includes polymer-based and hydrogel-based dressings with the therapeutic potential of bioactive molecules. Despite the significant development of techniques for creating dressing materials and the variety of types of the materials themselves, the effectiveness of such dressings, as well as the simplicity of their immediate use during war or in a combat environment, and therefore wound management still needs to be improved.

This section is aimed at characterization of commonly used simple hemostatic dressings in the management of battlefield wounds of various types.

##### QuikClot^®^ Combat Gauze and Celox™

Hemostatic dressings such as the kaolin-impregnated QuikClot^®^ Combat Gauze and chitosan-based Celox™ became standard in military field kits, owing to their rapid action, portability, and ease of use under combat conditions. These materials are non-toxic, require no preparation, and operate effectively even in coagulopathic patients.

QuikClot^®^ is a kaolin-impregnated rayon/polyester hemostatic dressing that was designated by the U.S. Armed Forces as the primary hemostatic management for the treatment of severe hemorrhage [44,103]. Indeed, the main mechanism resulting in hemostasis following the use of QuikClot is the absorption of water and the rapid concentration of platelets and clotting factors XII (FXII) and XI (FXI) of the intrinsic coagulation pathways that further catalyze a rapid clot formation [104,105]. As a result of its action, an effective reduction in the risk of major bleeding complications can be noticed. In addition, the hazard of delayed bleeding in patients with complicated peripheral arterial occlusive disease (PAOD) and comorbidity is obtained [103].

Apart from the above-mentioned, QuikClot^®^ produces a robust clot that can withstand more movement than a control dressing, as demonstrated in a Yorkshire swine in vivo model [106]. However, one notable side effect of QuikClot^®^, particularly reported in the case of the older type of QuikClot, i.e., zeolite-based, is the exothermic reaction that occurs when it comes into contact with moisture, such as blood. This reaction causes a rapid rise in temperature, typically reaching 42 °C to 44 °C, although Wright et al. [107] reported zeolite-based QuikClot^®^ to produce temperatures over 100 °C in the femoral vein, muscle, liver and skin and of 93.1 and 95.4 °C on the surface of the spleen and femoral artery, respectively, in a multiple injury model in swine. Although this thermal phenomenon persists for only a few minutes, it has been shown to result in localized tissue burns in several preclinical animal studies and in human subjects [43,107,108]. In turn, this caused mild to severe pain and discomfort. However, researchers have consistently concluded that the benefits of rapid hemorrhage control and increased survival far outweigh the risk of thermal tissue injury, especially in cases of severe, otherwise uncontrollable bleeding where immediate hemostasis is critical [109]. Nevertheless, the second generation of QuikClot, the so-called QuikClot ACS, produced from larger beads of the same composition as QuikClot, and packaged into mesh bags, was claimed to produce suppressed exothermia [43].

QuikClot^®^ demonstrated high effectiveness in field use by first responders across both civilian and combat settings. During military operations, particularly among U.S. Army medics and Navy corpsmen serving in the Iraq War, the product was most popular when applied to extremity wounds in hypotensive patients. As reported by Rhee et al. [108], in all such instances, QuikClot^®^ was employed to manage life-threatening hemorrhages.

The overall efficacy rate was reported to be high, with most failures attributed to cases involving moribund, coagulopathic patients in whom the dressing was applied as a last resort or improperly. In the latter case, it is believed that the nature of the wounds made it difficult to apply the product directly to the source of bleeding, thereby limiting its effectiveness in achieving hemostasis [43]. Despite these challenges, QuikClot^®^ remains a critical tool in emergency hemorrhage control, especially when applied promptly and directly to the bleeding site.

Celox™ is a hemostatic agent derived from chitosan, a biopolymer obtained from chitin, which is non-toxic, odorless, and biocompatible. It promotes rapid clot formation through a mechanism involving adsorption, dehydration and electrostatic interaction between its positively charged molecules and negatively charged red blood cells. This interaction facilitates rapid clot formation at the site of bleeding, functioning independently of the body’s intrinsic coagulation pathways [110,111]. Importantly, unlike QuikClot^®^, Celox™ does not produce an exothermic reaction, thereby minimizing the risk of thermal injury to surrounding tissues [112,113,114]. Moreover, in two independent animal studies by Kozen et al. [110] and by Littlejohn et al. [115] Celox™ was found to be superior to other commercial agents, as a greater reduction in total blood loss, lower incidence of re-bleeding and a significant decrease in mortality rates were noted. These preclinical findings strongly support the hemostatic potential of Celox™ in high-pressure vascular injuries. Notably, these actions were repeated during clinical observations in human subjects [111].

##### Axiostat Z-fold Hemostatic Gauze

**Figure 2:**
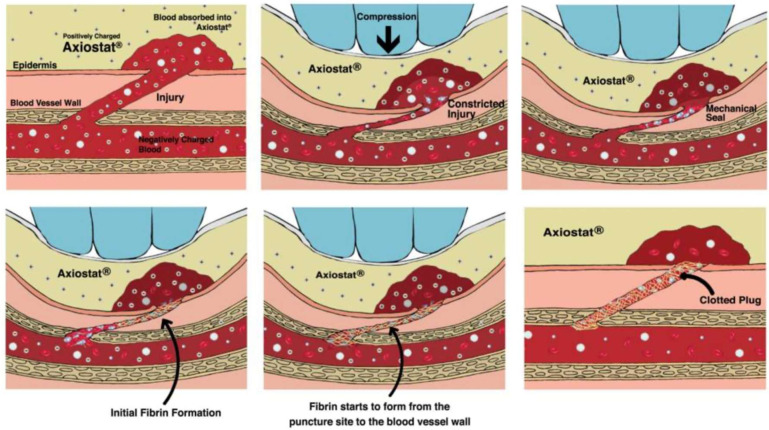
Mechanism of acition of Axiostat^®^ [from M. Kabeer, P.P.Venugopalan, Subhash VC, Pre-hospital Hemorrhagic Control Effectiveness of Axiostat^®^ Dressing Versus Conventional Method in Acute Hemorrhage Due to Trauma, Cureus. 29 August 2019;11(8):e5527 [119] under a CC-BY 4.0 commercial international license; http://creativecommons.org/licenses/by/4.0/; Accessed on 13 May 2025].

Axiostat^®^ is a pH-responsive, 100% chitosan dressing designed to stop bleeding instantly. In fact, owing to a highly porous chitosan matrix, it causes rapid absorption of plasma from the blood, which leads to accumulation of erythrocytes and platelets at the injury site (Figure 2). Simultaneously, the gauze’s high-water absorption slows the blood flow, making it denser [116]. Importantly, when Axiostat^®^ is applied with pressure to damaged tissue, its porous chitosan-based structure facilitates formation of mechanical interlocking with the tissue surface. This process creates microspaces within the pores that allow for instantaneous bioadhesion. Simultaneously, surface-bound components such as red blood cells and platelets diffuse into the internal pore network of the dressing, enhancing clot formation and achieving rapid hemostasis through both physical adhesion and biological interaction [117,118].

In one of the clinical studies presented by Kabeer and colleagues [119], hemostasis by using Axiostat^®^ was significantly lower than that of the conventional cotton gauze group (4.68 ± 1.04 min and 18.56 ± 5.04 min). Moreover, there was about a 50% reduction in blood loss from the wound site in cases of Axiostat^®^-treated patients as compared to that of the control group (13.70 g and 4.80 g for cotton gauze and Axiostat^®^, respectively). Other benefits of those dressings include the ability to use them for deep or irregular wounds owing to their Z-fold design that allows for controlled application and absence of the risk of thermal injury to tissues, as Axiostat does not produce heat upon application [120]. However, in the latter case, some studies indicated Axiostat to induce radial thrombosis, particularly in patients undergoing transradial coronary angioplasty [121].

##### Rev Medx’s xSTAT Hemostatic Agent

XStat™ is a first-in-class, FDA-approved hemostatic device specifically designed for the rapid control of life-threatening hemorrhage resulting from deep, non-compressible wounds, such as those caused by gunshot or shrapnel injuries on the battlefield. Unlike conventional gauze dressings, XStat employs a syringe-style applicator to deliver numerous miniaturized, rapidly expanding sponges directly into the wound cavity. Once they come in contact with liquid, they quickly expand and create an internal compression [122,123]. Importantly, in one preclinical study by Cox and Rall [124], XSTAT turned out to be better than other hemostatic wound dressings, in particular QuickClot^®^. Indeed, application of this unique type of dressing to animals (a swine model of lethal junctional hemorrhage) resulted in fast and prolonged homeostasis. Also, less blood was lost during the first 10 min after injury in the XSTAT group than in the QuickClot^®^-treated group. Others indicated its significantly faster administration and the ability to absorb more blood [125].

### 3.2. Next-Generation Multifunctional Wound Dressings That Offer Added Benefits

Currently, much work is being conducted in order to develop wound dressings that provide additional benefits for the patient. In this aspect, most of the proposed dressing materials combine different components to offer a range of benefits, including antimicrobial protection, pain-relieving effect, improved breathability, etc. [126].

#### 3.2.1. Wound Dressings with Antimicrobial Agents

Antimicrobial drugs are a useful tool to inhibit the infection induced by both fungi and bacterial strains. It seems of great importance, as several bacterial strains, such as *Pseudomonas* and *Staphylococcus* species, are known for their significant virulence, a number of deleterious consequences (e.g., sepsis and respiratory infection development [127]), and the ability to rapidly develop drug resistance [128,129].

Silver is one of the best-established molecules with antimicrobial and bactericidal properties. Currently, apart from silver ions (Ag+) in the form of silver salts, silver nanoparticles (AgNPs) have also been widely studied [130,131]. Silver exhibits biocompatibility and non-toxicity to human cells at low concentrations [132]. An important advantage is that silver ions have a relatively low toxicity to human cells while adversely affecting bacteria and fungi by interacting with bacterial cell membranes, inhibiting reproduction of harmful bacteria [133]. Unfortunately, today many bacteria can exhibit mechanisms of bacterial resistance to silver [134,135]. Indeed, several studies indicate that such an activity can be caused by the ability of bacteria either to aggregate silver particles or to reduce silver, thus resulting in the inhibition of silver contact with the cell [136]. However, the mechanism of silver resistance appears to be more complex. In addition to the aforementioned characteristics, silver has also been found to impair healing by exerting toxic effects on keratinocytes and fibroblasts, in particular when administered at high concentrations [137,138].

As for austere and combat environments, silver-based dressings have gained particular relevance owing to their antimicrobial efficacy and the ease of use. One such example is the Silverlon^®^ Combat Medic Wound Dressing, an elastic bandage composed of nylon fabric coated with pure metallic silver. This dressing demonstrated clinical effectiveness in managing a variety of complex injuries, including open fractures, blast and burn injuries, and traumatic amputations [139,140]. According to Aurora et al. [139], the use of this silver-nylon dressing was associated with a trend toward reduced wound infection rates (5.4 vs. 9.5%) even in full-thickness burn injuries, without a significant increase in burn-related complications. During military operations in Afghanistan, Silverlon dressings were applied in the treatment of mine-related and thermal injuries, often producing beneficial effect within a 7-day application window [141,142]. Antimicrobial activity has been documented against a range of common pathogens, including methicillin-resistant *Staphylococcus aureus* (MRSA), *Pseudomonas* and *E. coli* [143,144]. Notably, in addition to its antimicrobial properties, the dressing has been reported to contribute to pain reduction and accelerated wound healing [145]. However, when talking about its antimicrobial activity, emerging data indicate a shift in the microbial landscape among combat-related burn patients. In fact, traditional pathogens such as *Staphylococcus aureus* and *Pseudomonas aeruginosa*, are increasingly being supplanted by multi-resistant *Acinetobacter* and *Klebsiella* species, especially in burn patients involved in combat operations [146]. This evolving resistance pattern underscores the need for additional studies to evaluate the efficacy of silver-based dressings against these more resilient microorganisms in combat and post-combat clinical scenario.

Silver has also been employed in the form of nanocrystalline silver dressings, such as Acticoat^TM.^ A study evaluating the effects of this dressing on wound microbiology and wound healing outcomes was presented by Fries et al. [147]. Unfortunately, the used nanocrystalline silver dressing in respect to prevention was found without any beneficial score in the aspect of wound colonization or promoting healing; this type of dressing did, however, appear to significantly reduce malodor, a common and distressing symptom associated with battlefield wounds. Importantly, this study was carried out among patients who had sustained an injury requiring wound debridement and treated at a military medical facility in Afghanistan. The specificity of the population and clinical setting lends weight to the findings and underscores the practical relevance of Acticoat™ use in combat-related trauma care.

**Figure 3:**
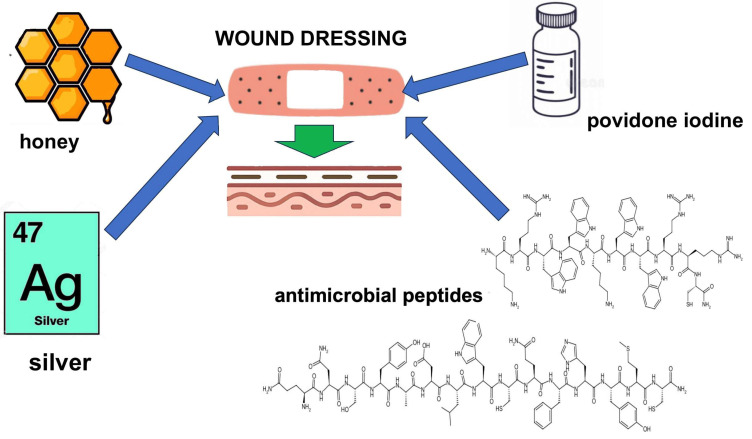
Examples of antimicrobial agents that serve as wound dressing active components.

Honey has been proposed as an antibacterial agent useful in the treatment of infections over a wide range of wound types. In line with this, Manuka honey (Figure 3) dressings were proposed to be effective owing to the high osmolarity created by their sugar content [148,149,150]. In addition, a promotion of wound healing infected with antibiotic-resistant bacteria such as MRSA has been reported [151]. However, in a study by Guthrie and colleagues [152], a Manuka honey dressing (Activon Tulle) had poor antibacterial activity in an animal model (New Zealand rabbit) of an *S. aureus* contaminated extremity muscle injury. This is in agreement with a well-established opinion that this brand of honey works well only when it is used along with antibiotics [149].

Other brands of dressing with potent antimicrobial properties include povidone-iodine and cadexomer iodine formulations, for which numerous studies reported utility [153,154,155]. However, evidence suggests that iodine-based dressings could be less effective as compared to other commercial wound dressings. For instance, a meta-analysis by Zhang et al. [156] revealed that honey-based dressings were associated with improved wound healing outcomes as compared to those based on povidone iodine. Similar findings were provided by Jiang et al. [157], who reported that silver-containing dressings significantly reduced wound healing time as compared with those of iodine-based alternatives. Moreover, it should be emphasized that the use of iodine-based dressings imposes certain limitations and contradictions. Specifically, their application can be restricted to patients with: (i) iodine sensitivity, as iodine can induce toxicity, particularly contact-irritant dermatitis [158]; (ii) thyroid disorders, due to systemic absorption of iodine [154]; (iii) wounds with substantial depth or complex anatomical structure [159].

Among various brands of antimicrobial agents used in wound dressings, antimicrobial peptides (AMPs) have gained much attention. Indeed, in addition to human antimicrobial peptides such as human beta-defensin (hBD) and LL-37, thousands of natural AMPs have been identified across a wide range of species, including invertebrates, mammals, microbes, and plants [160]. Despite their promising biological characteristics, there remains limited data on the development and implementation of AMP-based wound dressings in battlefield settings. Nonetheless, recent advances in AMP engineering and delivery systems can soon enable their application in military medicine. A selection of representative AMP-based wound dressing strategies is presented below to illustrate their potential.

In 2024, Chen et al. [161] developed a novel multifunctional wound dressing (PVA-TPE/HA-AMP/SF/ALG) composed of polyvinyl alcohol (PVA), hyaluronic acid (HA) conjugated with an antimicrobial peptide (AMP) featuring the sequence KRWWKWWRRC (Figure 3), silk fibroin (SF), alginate (ALG), and a tetraphenylethylene (TPE)-based aggregation-induced emission (AIE) component. This advanced composite dressing showed a strong antimicrobial effect against *S. aureus* and *E. coli*. Moreover, it demonstrated a significant enhancement in tissue regeneration, attributed to its ability to promote cell proliferation and stimulate wound neovascularization, as confirmed through both in vitro and in vivo studies.

Another example shows a polyurethane dressing coated with a thrombin-derived, TCP-25 (GKYGFYTHVFRLKKWIQKVIDQFGE; Figure 3) [162]. This peptide-functionalized hydrophilic foam dressing yielded a rapid reduction in *P. aeruginosa* and *S. aureus* bioluminescence already after 1 min of incubation in vitro. Moreover, this antimicrobial effect was repeated in a mouse model (BALB/c male mice) of subcutaneous infection.

Jia et al. [163] demonstrated recently developed a photothermal antibacterial composite hydrogel (MNPs/CyRL-QN15/FeCMCS) fabricated with a coating of Fe^2+^cross-linked carboxymethyl chitosan (FeCMCS) following incorporation of melanin nanoparticles (MNPs) and the cyclic derivative of the 15-amino-acid peptide RL-QN15 (primary sequence QNSYADLWCQFHYMC; Figure 3), originally identified from *Rana limnocharis* skin secretions [164], CyRL-QN15. The hydrogel formulation significantly promoted proliferation and migration of both keratinocytes and fibroblasts, key cellular events in wound re-epithelialization and matrix remodeling. In addition to its regenerative properties, the hydrogel exhibited potent antibacterial activity, effectively eliminating over 95% of both Gram-positive and Gram-negative bacteria. In vivo studies conducted using an MRSA-infected full-thickness wound animal model further confirmed its therapeutic potential. Treatment with the hydrogel not only reduced the inflammatory response but also accelerated wound re-epithelialization and enhanced collagen deposition, thereby facilitating the regeneration and remodeling of chronically infected tissue.

#### 3.2.2. Wound Dressings with Pain-Relieving Agents

In addition to the risk of widespread wound contamination, various types of injuries are invariably associated with pain. Notably, pain experienced during dressing changes is often reported as one of the most distressing aspects of living with a chronic wound [165]. However, pain also exists as a result of the wound itself due to skin damage, nerve damage, blood vessel injury, infection and ischemia [166]. Therefore, dressings with incorporated analgesics are highly desired. Although there are many reports on the topical administration of analgesics in the form of, for example, creams, there is little information on the design of dressings that would additionally contain an analgesic drug in their formulation. Nevertheless, some of the existing findings are given below.

Recent studies from 2025 have demonstrated that a dressing loaded with a non-opioid analgesic, ropivacaine, is effective in reducing pain sensation in partial-thickness dermal wounds [167]. The tested dressing comprises a biodegradable matrix made from poly(lactide-co-caprolactone) (PLCL), which facilitates controlled release of ropivacaine. The incorporation of ropivacaine into wound dressings allows for sustained, localized analgesia directly at the wound site, minimizing the need of systemic analgesics and enhancing patient’s comfort during wound care. In comparative analyses, ropivacaine-loaded dressings demonstrated superior pain control over conventional non-medicated dressings, especially during dressing changes and routine wound management procedures. Moreover, owing to the sustained release, it exhibited a pain-relieving effect over a 168 h period as compared to that of a control silicone dressing.

Aycan et al. [168] developed a sodium alginate/gelatin/hyaluronic acid/reduced graphene oxide (SAlg/Gel/HA/RGO) electroconductive composite film-based wound dressing and demonstrated that ibuprofen-loaded variants exhibited significantly enhanced therapeutic efficacy as compared to drug-free loaded ones.

Ibuprofen was also investigated by researchers such as Sibbald et al. [169] and Jørgensen et al. [170]. In the first study conducted at a Canadian wound clinic involving 24 patients with chronic leg ulcers, the authors reported that application of an ibuprofen-impregnated foam dressing resulted in a significantly enhanced reduction in wound-related pain as compared to that of standard local wound care practices, which included moist healing dressings and antimicrobial or anti-inflammatory alternatives [169]. Similar findings were reported using a commercial Biatain-Ibu Non-Adhesive Coloplast A/S (Denmark)—a soft, conformable polyurethane foam dressing impregnated with ibuprofen (0.5 mg/cm^2^) [170,171].

Despite the satisfactory results, the use of non-steroidal anti-inflammatory drugs (NSAIDs), such as ibuprofen and diclofenac, in wound dressings remains controversial. This divergence of opinion concerns their anti-inflammatory rather than analgesic properties. In fact, while some studies suggest NSAIDs-releasing dressings to support wound healing by attenuating excessive inflammation and stimulating tissue regeneration [172], others highlight concerns that suppression of the early inflammatory phase could disrupt physiological repair mechanisms, thereby delaying the healing process [173]. Nevertheless, a study conducted by Costa et al. [174] showed no adverse impact on wound healing following the topical application of diclofenac gel.

As certain injuries often require stronger analgesic management, opioids remain a first-line therapeutic option. In this context, a Polish research group led by Kowalski et al. [175] characterized sufentanil-based dressing applied postoperatively in burn wound treatment. The use of opioid drugs in thermal wounds is well-justified, as opioids are known to alleviate both pain and inflammation—processes commonly associated with burn trauma. This effect is largely attributed to activation of peripheral opioid receptors [176,177,178,179]. Hence, there is a low risk of inducing clinically significant side-effects resulting from central opioid actions, especially with compounds that do not readily cross the blood–brain barrier. Nevertheless, in the referenced study, a three-layer sterile dressing was employed consisting of an inner layer soaked with a solution of octenidine hydrochloride, phenoxyethanol, and sufentanil (concentrations of 5, 25 and 50 µg); a middle paraffin-impregnated layer; and an outer dry protective layer. This type of dressing demonstrated a dose-dependent analgesic effect, significantly reducing pain in human subjects following burn surgery without the overall need for rescue analgesics and the occurrence of common opioid-related systemic side-effects such as sedation, nausea and respiratory depression [175].

The same group explored the analgesic effect of a wound dressing embedding morphine [180] in a prospective, double-blinded, randomized controlled clinical trial. Again, the specific dressing composition was soaked with a solution of morphine sulfate at concentrations of 10 mg or 20 mg, along with octenidine and phenoxyethanol. As a result, Kowalski et al. reported morphine to be an effective analgesic in patients after surgical debridement of burn wounds with the pain intensity Numeric Rating Scale (NRS) value equal to 0 for patients treated with the higher dose of morphine.

Intriguingly, among opioid-like compounds used, attention should be given to biphalin, a dimeric enkephalin with a potent analgesic effect, synthesized by Lipkowski in 1982 [181]. Nevertheless, application of this peptide has not been aimed at relieving pain, though this is one of its principal features, but to accelerate wound closure; this effect has widely been attributed to opioids and the opioidergic system [182,183,184]. In this context, biphalin incorporated in a mouse fur keratin-based dressing was found to be effective in a mouse model of diabetic wounds [185], as a tendency for faster healing was reported. However, no statistical significance was provided.

While analgesic-loaded wound dressings offer promising localized pain relief and improve patient comfort, their long-term use raises several important considerations. One primary concern is the possibility of systemic absorption of the analgesic agents, especially when dressings are applied over large wound areas, chronic wounds, or damaged skin barriers. Prolonged exposure can lead to cumulative systemic uptake, which may result in unintended side effects, toxicity, or drug interactions, particularly with opioids or NSAIDs. Intriguingly, some studies suggest that NSAIDs may not only impair wound healing [186,187], but also promote the attachment of opportunistic bacteria, such as group A *Streptococcus pyogenes* (GAS), to muscle tissue [188].

Additionally, regulatory challenges present a significant hurdle for the development and clinical adoption of such dressings. Combining medical devices with pharmacologically active substances blurs the line between device and drug categories, complicating approval pathways. Regulatory agencies require comprehensive safety and efficacy data, including long-term toxicity, pharmacodynamics, and potential systemic effects [189,190,191].

Finally, patient-specific factors such as comorbidities, concurrent medications, and wound characteristics must be carefully considered to tailor analgesic-loaded dressing use and avoid adverse outcomes. These limitations emphasize the need for thorough preclinical and clinical evaluation to ensure safe and effective use of analgesic-loaded dressings in wound care.

#### 3.2.3. Wound Dressings Containing Biological Factors

Considering that biological factors play a key role in the process of wound healing, affecting each of its stages, from hemostasis to inflammation, proliferation and tissue remodeling, efforts have been made to use them directly in wound dressings. While current literature does not provide evidence that such biologically active dressings are routinely employed in battlefield settings, existing data on their therapeutic potential strongly support their future implementation in trauma care. These findings can serve as a foundation for initiating clinical translation and adapting advanced biological dressings for use in military and austere environments.

The crucial features are growth factors (GFs), such as platelet-derived growth factor (PDGF), vascular endothelial growth factor (VEGF), epidermal growth factor (EGF), transforming growth factor-beta (TGF-β), and fibroblast growth factor (FGF) [192]. Each plays a critical role in tissue regeneration and healing process, although at different steps. For example, VEGF is the principal driver of angiogenesis and is produced by keratinocytes, fibroblasts, and inflammatory cells in response to hypoxia and injury. It stimulates endothelial cell proliferation and migration and increases vascular permeability, facilitating nutrient and immune cell delivery to the wound site [193]. VEGF is especially critical during the transition from the inflammatory to the proliferative phase of healing [194]. In contrast, EGF, synthesized by keratinocytes, macrophages, and platelets, plays a vital role in re-epithelialization by stimulating the proliferation and migration of keratinocytes [195]. While PDGF, primarily secreted by activated platelets and macrophages, acts as a potent chemoattractant for fibroblasts, smooth muscle cells, and neutrophils. It promotes fibroblast proliferation and ECM production, while also indirectly supporting angiogenesis [196].

Taken together, these growth factors play a pivotal role in orchestrating the wound-healing cascade and have emerged as highly promising targets for therapeutic intervention. Their incorporation into contemporary wound care approaches, such as topical delivery platforms, sustained-release systems, and tissue-engineered scaffolds, has opened new frontiers in regenerative medicine, enabling more effective and biologically guided strategies for tissue repair and restoration.

Regranex^®^ Gel (Becaplermin 0.01%, Smith & Nephew, UK), a PDGF-BB (platelet-derived growth factor)-based topical agent, represents a key example of the growing trend toward the incorporation of biological factors into wound dressings for the treatment of wounds, particularly diabetic foot ulcers [197]. This growth factor-loaded formulation is designed to stimulate cellular proliferation and promote granulation tissue formation, thereby enhancing the healing process.

However, the biological activity of growth factor-based treatments can be variable and difficult to predict, especially outside the controlled environment of clinical trials. For instance, it is well-known that high levels of proteolytic activity in vivo lead to poor GFs stability and short half-life [198]. In addition, in the case of Regranex^®^, post-marketing surveillance revealed certain limitations in efficacy, particularly in infected or ischemic wounds, as well as safety concerns. Notably, subsequent evaluations indicated a potential increased risk of malignancy associated with prolonged or excessive use—an outcome not initially evident in pre-approval clinical studies [199,200].

Nevertheless, the interest in GFs utility in wound dressings persists. For example, Chakrabarti et al. [201] demonstrated a basic fibroblast growth factor (bFGF) incorporating silver sulfadiazine hydrogel formulation (bFGF-collagen-AgSD). The use of bFGF is well-argued by the fact that this prominent member of the heparin-binding protein family, plays a crucial role in regulating the proliferation, differentiation, and migration of various cell types. It serves as a key modulator in normal tissue development, maintenance, and repair, and is especially important in wound healing and angiogenesis [202,203,204]. Through its broad biological activity, bFGF contributes significantly to cellular function and tissue regeneration, making it a valuable target in therapeutic and biomaterial-based approaches to regenerative medicine. In this aspect, the newly developed bFGF-collagen-AgSD hydrogel dressing was found to facilitate rapid burn wound healing while simultaneously preventing pathogenic infections in an animal model of partial thickness burn [204].

Also, Asiri et al. [205] demonstrated the potential utility of growth factors (GFs) in advanced wound dressings through the development of multilayered polyvinyl alcohol (PVA) electrospun nanofiber scaffolds incorporating epidermal growth factor (EGF) and fibroblast growth factor (FGF). This GF-loaded nanofiber system was shown to enhance cell proliferation without inducing cytotoxic effects, highlighting its biocompatibility. Notably, the multilayer PVA-GF nanofiber scaffolds resulted in a significant wound size reduction by day 7 and improved tissue repair by days 14 to 21, as compared not only to that of the untreated controls but also to single-layer PVA-GF nanofiber constructs. These findings underscore the synergistic effect of combining multiple growth factors within a multilayered nanostructured delivery system for accelerated wound healing.

In contrast, Yoshikawa et al. [206] demonstrated topical delivery of bone marrow-derived mesenchymal stem cells (MSCs) via a collagen sponge scaffold that significantly promoted wound healing, with clinical improvement recorded in 18 out of 20 patients.

As just presented, recent advances in regenerative medicine have led to the development of next-generation wound dressings that either deliver exogenous growth factors or incorporate stem cells capable of endogenously producing them. Growth factor-releasing dressings are engineered to provide targeted and localized delivery of recombinant proteins, thereby enhancing cellular activation, promoting angiogenesis, and accelerating tissue regeneration at the wound site. In parallel, stem cell-based dressings, particularly those incorporating mesenchymal stem cells (MSCs) or amniotic-derived tissues, exert multifaceted effects through the paracrine secretion of bioactive molecules with both regenerative and immunomodulatory properties. These innovative materials hold considerable promise for improving outcomes in complex or non-healing wounds, especially those unresponsive to conventional therapies.

Taken together, these innovative strategies represent a critical evolution in combat wound care. Unlike traditional dressings that provide passive protection, next-generation multifunctional materials can intervene directly in the healing cascade—by reducing inflammation, suppressing infection, promoting angiogenesis, and relieving pain simultaneously. This proactive and integrative approach may substantially improve survival, reduce complications, and enable earlier functional recovery in military personnel injured under austere conditions.

However, despite these encouraging developments, the clinical translation of growth factor- and stem cell-enhanced dressings into battlefield wound care remains constrained by several critical challenges. One of the foremost limitations is the biological instability of growth factors [207]. These proteins are highly susceptible to proteolytic degradation in the wound environment and demonstrate short half-lives, which compromises their therapeutic effectiveness. Moreover, their temperature sensitivity complicates storage and transport, making cold-chain logistics a prerequisite—an impractical requirement in austere military environments [208,209].

Similarly, stem cell-based formulations face serious hurdles related to cellular viability and functionality under field conditions. Mesenchymal stem cells (MSCs), while promising in vitro and in clinical trials, are vulnerable to hypoxia, oxidative stress, and mechanical disruption, all of which can occur during storage, deployment, or application in combat scenarios. Additionally, inter-donor variability and immunogenicity concerns further complicate the development of standardized, universally applicable products [210,211,212,213].

Scalability is another barrier. The large-scale production of biological factors-based dressings (e.g., GF- or MSC-integrated dressings) under good manufacturing practice (GMP) conditions remains cost-intensive and technologically demanding [214]. Moreover, regulatory considerations must not be overlooked. Products containing viable cells or recombinant growth factors are typically classified as advanced therapy medicinal products (ATMPs) and are subject to extended safety, efficacy, and quality evaluation by agencies such as the EMA or FDA [215,216]. These regulatory hurdles considerably delay clinical implementation, particularly in time-sensitive military applications.

In conclusion, while GF- and stem cell-based dressings represent a transformative approach in regenerative wound care, their effective implementation in military medicine will depend on overcoming substantial biochemical, logistical, and regulatory barriers. Future efforts should focus on enhancing the robustness, shelf-stability, and ease of application of these advanced systems to meet the unique demands of combat casualty care.

## 4. Conclusions

The management of combat-related wounds remains one of the most challenging aspects of military medicine due to the complex, contaminated, and often life-threatening nature of these injuries. Traditional wound care approaches are frequently inadequate in battlefield environments, where time, resources, and specialized personnel are limited. Recent advances in the development of multifunctional wound dressings have demonstrated significant potential to address these limitations by integrating antimicrobial, hemostatic, and analgesic properties within a single, easy-to-apply format.

This review highlights the therapeutic value of innovative dressing materials that incorporate both synthetic and biologically active components—such as silver, NSAIDs, opioids, and natural polymers—which collectively enhance wound healing outcomes in austere conditions. In particular, opioid- and NSAID-loaded dressings have shown promising analgesic efficacy while minimizing systemic side effects, and silver-based formulations remain a cornerstone of infection control, despite emerging microbial resistance. Moreover, the inclusion of bioactive substances like collagen, thrombin, chitosan, alginate, and medicinal plant extracts offers additional regenerative and hemostatic benefits, as demonstrated in both preclinical and clinical studies. However, the effectiveness of these materials is variable depending on the wound type, injury mechanism, and anatomical location—factors that must be carefully considered in dressing selection.

Despite the progress made, further research is needed to optimize the balance between therapeutic efficacy and safety, particularly regarding the inflammatory modulation and long-term outcomes of bioactive dressings. Indeed, future research should prioritize the development of dressing systems with sustained drug release, improved biocompatibility, and field-tested durability. Additionally, translational studies addressing scalability, cost-effectiveness, long-term safety, and regulatory approval pathways are critical to bringing multifunctional dressings from bench to battlefield.

As the nature of warfare continues to evolve, so too must the strategies for managing combat trauma. The development of next-generation, multifunctional dressings tailored for battlefield use represents not only a technological advancement but also a critical step towards improving survival and quality of life for injured personnel.

## Figures and Tables

**Table 1:** Classification of war wounds made by the Red Cross.

Wound Classification	Wound Size		Types of Wounds			
	Entry/Exit Wound Diameters; E + X [cm]	Cavity Diameter; C [Finger]	Soft Tissue Injury (ST)	Fracture Type; F	Critical Organ Injury; V	Critical Organ Injury + Fracture; VF
Grade I—low-energy transfer	<10	Less than 2	Small, simple	1	1	1
Grade II—high-energy transfer	<10	>2	2	2	2	2
Grade III—massive wounds	>10	>2	3	3	3	Large wounds that can be life-threatening or that can damage limb functions

Grade I includes the so-called low-energy transfer wounds, which means that soft tissue injury is confined to the track of the projectile. In contrast, in the case of high-energy transfer wounds (Grade II), soft tissue injury is confined to the track of the projectile in addition [3]. F is for fracture; F = 0—no fracture; F = 1—simple fracture, hole or insignificant comminution; F = 2—clinically significant comminution. Type V is for vital structure and includes central and peripheral wounds such as injuries of the brain or spinal cord, of major peripheral blood vessels, etc.

**Table 2:** Comparative properties of common dressing materials used in combat settings.

Material	Absorptive Capacity	Biodegradability	Hemostatic Potential
Chitosan	Moderate	High	High
Kaolin	High	Non-biodegradable	High
Alginate	High	High	Moderate to high
Zeolite	High	Non-biodegradable	High (with exothermic risk)

**Table 3:** Selected examples of the effectiveness of hemostatic dressings in various animal models of wounds not presented in the main text.

Hemostatic Dressing Type (Including Type of the Active Ingredient)	Wound Type/Model	Animal	Outcomes	Ref.
Topical hemostatic dressings with one of the ingredients: microfibrillar collagen, oxidized cellulose, thrombin, fibrinogen, propyl gallate, aluminum sulfate, and fully acetylated poly-N-acetyl glucosamine	a model of severe venous hemorrhage and hepatic injury	Yorkshire swine	hemostatic dressing that contains fibrinogen or thrombin reduced posttreatment blood loss	[51]
Lyophilized, ready-to-use fibrin-based sheets	rabbit ear artery	rabbit	immediate stop of bleeding (in 3–5 s)	[94]
Fibrin bandage	ballistic (gunshot; 0.308 caliber high-velocity bullet) wound	Angora goats	significant reduction in the blood loss; the mean arterial pressure was also maintained higher	[95]
The mineral zeolite powder QuikClot	lethal groin injury model	Yorkshire swine	increased survival and decreased hemorrhage in comparison to the untreated control group	[49]
Polivynyl alcohol (PVA)/sodium alginate (SA) hydrogel loaded with nitrofurazone	excision of dorsum (two full thickness skin wounds of 1.5 cm × 1.5 cm area)	Male Sprague Dawley rats	wound size reduction with new epithelium noted at the edge of the defect. However, overall, the positive healing effect was similar to that with PVA only	[96]
Commonly used QuikClot Combat Gauze, ChitoGauze, NuStat Tactical, Kerlix with no clotting agent	a unilateral arterial hemorrhagic groin injury (a swine model of prolonged field care with limb movement)	Swine	combat Gauze proved to have the lowest incidence of rebleeding, while the NuStat Tactical (made of regenerated cellulose and silica-based fibers) had the highest incidence of rebleeding at wounds after limb movement	[97]
Woven fiber matrix made from regenerated cotton cellulose, BloodSTOP iX Battle Matrix	swine extremity arterial hemorrhage model	Yorkshire swine	longer survival and significantly shorter times to hemostasis as compared to animals treated with QuikClot Combat Gauze	[98]
Algan Hemostatic Agent (AHA)	the femoral artery damage model	Rats	a significantly shorter time of bleeding compared to that of the control	[99]

## Data Availability

No new data were created or analyzed in this study.
